# Intra-specific comparison of mitochondrial genomes reveals host gene fragment exchange via intron mobility in *Tremella fuciformis*

**DOI:** 10.1186/s12864-020-06846-x

**Published:** 2020-06-24

**Authors:** Youjin Deng, Xunxiao Zhang, Baogui Xie, Longji Lin, Tom Hsiang, Xiangzhi Lin, Yiying Lin, Xingtan Zhang, Yanhong Ma, Wenjing Miao, Ray Ming

**Affiliations:** 1 Center for Genomics and Biotechnology, Haixia Institute of Science and Technology, College of Life Sciences, Fujian Agriculture and Forestry University, Fuzhou, 350002 China; 2 Department of Plant Biology, University of Illinois at Urbana-Champaign, 1201 W. Gregory Drive, Urbana, IL 61801 USA; 3 Environmental Sciences, University of Guelph, Guelph, ON N1G 2W1 Canada

**Keywords:** *Tremella fuciformis*, Mitochondrial genome, Intron with N-terminal duplication, Intron mobility

## Abstract

**Background:**

Mitochondrial genomic sequences are known to be variable. Comparative analyses of mitochondrial genomes can reveal the nature and extent of their variation.

**Results:**

Draft mitochondrial genomes of 16 *Tremella fuciformis* isolates (TF01-TF16) were assembled from Illumina and PacBio sequencing data. Mitochondrial DNA contigs were extracted and assembled into complete circular molecules, ranging from 35,104 bp to 49,044 bp in size. All mtDNAs contained the same set of 41 conserved genes with identical gene order. Comparative analyses revealed that introns and intergenic regions were variable, whereas genic regions (including coding sequences, tRNA, and rRNA genes) were conserved. Among 24 introns detected, 11 were in protein-coding genes, 3 in tRNA genes, and the other 10 in rRNA genes. In addition, two mobile fragments were found in intergenic regions. Interestingly, six introns containing N-terminal duplication of the host genes were found in five conserved protein-coding gene sequences. Comparison of genes with and without these introns gave rise to the following proposed model: gene fragment exchange with other species can occur via gain or loss of introns with N-terminal duplication of the host genes.

**Conclusions:**

Our findings suggest a novel mechanism of fungal mitochondrial gene evolution: partial foreign gene replacement though intron mobility.

## Background

Parasitism is one of the most intricate phenomena in biology. Generally, parasitism is a non-mutualistic relationship between species, where the parasite reduces the biological fitness of the host, while it increases its own fitness by obtaining resources necessary for survival from the host. The relationship between mobile elements and their host genomes is also referred to as a type of parasitism at the genomic level [1–3]. A mobile element is a DNA sequence that can change its position within a genome or insert into another genome. It utilizes host cellular machinery for element duplication and mobility, but is traditionally regarded to have little or no benefit for the host [3, 4]. Different from nuclear introns, mitochondrial introns are typical selfish mobile elements [5].

Mitochondrial genome comparisons among isolates within a species or closely related species have revealed some extra-large fragments [5–13]. In most cases, these fragments range from several hundred bp to several kb in size, contain one intron-encoded protein gene (IEP), and are located between exons of a conserved gene, and hence referred to as introns. These fragments did not evolve from their own genome, but resulted from parasitism by mobile elements from other genomes. When their host genes start transcription, the introns act as ribozymes to remove their own sequences from the primary transcripts, thus limiting the impact on functionality of their host [1]. Sometimes, one intron is invaded by another intron to form a complex intronic structure, referred to as a twintron [14–17]. At least two levels of parasitism exist in this situation: relationships between parasite intron and host intron, and between twintron and host gene.

Based on the RNA secondary structure, introns in fungal mitochondrial genomes are classified into two major groups [18]. Group I introns generally encode a type of self-splicing ribozyme mostly containing 10 conserved helices and a conserved catalytic core [19], and spread widely through hosts by mobility and horizontal transfer. Two hypotheses are common to explain the mobility of group I introns. One hypothesis is intron homing based on the harbored homing endonuclease gene [19–21]. The recognition site of the homing endonuclease is located in a sequence with 14–45 nucleotides around a break point. The other hypothesis is intron invasion using an RNA intermediate for reverse splicing. According to this hypothesis, a 4–6 nt internal guide sequence is employed to recognize the target region through complementarity [22]. Group II introns are much less common in fungal mitochondrial genomes [5], where splicing occurs by two transesterification steps virtually identical to nuclear pre-mRNA splicing [23].

Recent studies provide evidence that mobility of introns may affect their host genes, including gene structure and DNA composition. The *Gigapora rosea cox1* gene is broken up into two fragments via group I intron-mediated trans-splicing. The two fragments are on the same strand in the mitochondrial genome, and are separated by a sequence of ~ 30 kbp, which includes 15 genes. Similar cases of group I intron-mediated trans-splicing have also been reported in the *cox1* gene in *Gigaspora margarita* [24], *Isoetes engelmannii* [25]*, Selaginella moellendorffii* [26]*, Helicosporidium* sp. [27]*,* and placozoan animals [28]*,* and in the *rns* gene in *G. margarita* [24]. A higher density of single nucleotide polymorphisms in exons near self-splicing introns was detected when analyzing the mitochondrial genomes of *Saccharomyces cerevisiae*, *Schizosaccharomyces pombe*, and *Lachancea kluyveri*, leading to the deduction that intron mobility is a direct driver of host gene diversity (Repar and Warnecke 2017). However, no evidence has been reported that gain and loss of introns can give rise to large fragment changes in host genes.

*Tremella fuciformis* Berk., a popular edible fungus in Asia, belongs to Tremellaceae (Tremellomycetes, Basidiomycota). This mushroom is in demand for medicinal use, such as the improvement of the immune system and anti-diabetic effects [29, 30]. In this study, we sequenced entire genomes of 16 *T. fuciformis* isolates using Illumina and PacBio sequencing technologies, and assembled them. We then pulled out mtDNA-related contigs and finished their assembly into complete mitochondrial molecules by more carefully examining the raw reads. Then we compared the mitochondrial genomes to investigate the types, locations and presence/absence of introns. We concentrated on the gain and loss of introns containing N-terminal duplication of the host genes. The overarching goal of this work is to investigate possible evolutionary pathways for mitochondrial protein coding genes.

## Results

### Comparisons of *T. fuciformis* mitochondrial genomes

Three different types of raw reads (100 bp, 125 bp and 250 bp pair-end) were generated from 16 strains of *T. fuciformis* using the Illumina HiSeq 2500 platform (Supplementary Table 1). Paired-end read numbers ranged from 7.13 × 10^6^ to 2.50 × 10^7^, totaling 2.68 Gb to 6.70 Gb of raw data, with coverage from 63.1 X to 172.9 X.

To further confirm sequence accuracy, two isolates, TF13 and TF15 were subjected to PacBio RS II sequencing. The raw data (3.55 × 105 and 4.87 × 105) were trimmed into 1.23 × 10^5^ to 2.04 × 10^5^ corrected reads, which had average lengths of 9.1 Kb and 8.1 Kb, respectively. The PacBio assemblies were compared with their respective Illumina assembly of the same isolate to correct and confirm the sequences.

Mitochondrial DNA of the 16 sequenced *T. fuciformis* isolates was circular with a length ranging from 35,104 bp of TF01 to 49,044 bp of TF05. The mtDNAs of TF02, TF03, TF04, TF10, TF13, and TF16 were identical in sequence, collectively referred to as TF04 series; TF11 and TF14 had same mtDNA sequences, known as TF11 series. A 46,314-bp mitochondrial contig with a repeat sequence at its two ends was isolated from the genome assembly of TF13 PacBio reads, which represented a 40,579-bp circular DNA sequence. Nine single-base indels were detected by aligning the contigs assembled by PacBio and Illumina reads. These indels included seven G, one T, and one C deletions. Similarly, a contig containing the whole mtDNA sequence of TF15 was also found in its assembled PacBio reads. Only one singleton indel difference was detected between mtDNA of TF15 from Illumina (40,104 bp) vs PacBio sequencing (40,103 bp). All the indels from TF13 and TF15 except one were determined in the areas of single-base repeat sequences. Sanger sequencing was used to sequence these polymorphic areas, and results were identical with the products obtained from Illumina sequencing data. In other words, all the indels come from sequencing or/and assembly errors of PacBio data.

All mitochondrial genomes harbored the same set of 41 conserved genes, including 15 protein coding genes (three subunits of ATP synthase, three cytochrome oxidase subunits, seven subunits of the NADH dehydrogenase, apocytochrome b and *rps3*), small and large ribosomal subunits (*rns* and *rnl*), an RNA component of the mitochondrial RNAse P (*rnpB*), and 23 tRNAs. Among these tRNA genes, nine were clustered into the area between *nad6* and *cox3*, four between *nad4* and *cob*, and the other 10 tRNA genes distributed in other areas. The tRNAs corresponded to all 20 standard amino acids except for *Cys*, four of which (*Leu*, *Met*, *Arg*, and *Ser*) had two tRNA isoacceptors, and the other 15 had one isoacceptor each. In the mtDNA of all isolates, 35 conserved genes were encoded on the same DNA strand, the other six, including *cox3*, *trnR*, *rps3*, *rpnB*, *trnM*, and *atp9*, were located on the opposite strand.

The overall GC content was similar for the 16 mtDNAs of *T. fuciformis* with an average of 37.89% (Supplementary Table 2). The intra-specific GC content of protein-coding genes, rRNAs, tRNAs, and intergenic region differed significantly (*P* < 0.01) from each other. The average GC content of intergenic regions (mean GC = 29.8%) was much smaller than that of other regions (mean GC > 39.0%). No significant differences in GC content were found between protein-coding genes and introns. Interestingly, mitochondrial genomes of *T. fuciformis* differed from that of *T. mesenterica* significantly not only in total GC percentage (average △GC = 9.26%), but also in GC content of conserved protein-coding genes (average △GC = 5.66%), rRNAs (average △GC = 5.85%), tRNAs (average △GC = 3.31%), introns (average △GC = 10.81%), and intergenic region (average △GC = 8.54%).

### Intra-specific diversity among different areas of mtDNAs

In order to investigate intra-specific diversity among the areas of protein-coding genes (first two base pairs of codons and third base pair of codons), tRNAs, rRNAs, and intergenic regions (rejecting mobile fragments), mutation rates between the areas of TF04 and corresponding areas of the other 15 isolates were calculated (Table 1). The mutation rates of intergenic regions, as well as the third position of codons for protein-coding genes were much higher than those of rRNAs, tRNAs, and the first two position of codons, indicating that intergenic regions were the most variable regions in the *T. fuciformis* mitochondrial genomes. The intergenic region sequences and that of the third position of codons had similar mutation rates. The sequences for the first two positions of codons underwent the least change. Using mtDNA of TF04 as a reference, the order for average variation rates of other isolates from low to high was as follows: TF12 < TF05 < TF09 < TF06 < TF07 < TF01 < TF15 < TF11 < TF08, which mainly corresponded to the phylogenetic tree based on fourteen conserved proteins (excluding *rps3*).

**Table 1:** Comparison of mitochondrial genomes of 16 isolates of *T. fuciformis* as well as *T. mesenterica* ATCC28783 obtained in this study

Isolates	Genome size	GC content	Intron size	Number of introns	Intergenic region^1^	Intergenic region^2^	SNPs/kb (10–3)				
							In CDS		rRNA	tRNA	Intergenic region^2^
							First two base pairs	Third base pair			
TF04	40,586	37.92	13,525	10	1864	5766	–	–	–	–	–
TF12	40,590	37.92	13,527	10	1864	5765	0.1	0	0	0	0.1
TF05	49,044	38.10	21,613	14	1864	6269	0.1	0.9	0	0.6	0
TF06	36,670	38.17	10,877	10	–	6373	2.5	48.9	5.8	7.8	56
TF07	38,983	37.85	13,734	11	–	5822	5.2	53.6	5.4	7.2	57.3
TF08	38,528	37.67	10,525	8	2684	5889	3.2	60.4	9.7	5	138.4
TF01	35,104	37.55	9730	7	–	5903	2.4	49.9	9.7	5	74
TF09	36,682	37.90	11,392	10	–	5854	2.5	41.6	4.9	7.2	26
TF11	40,338	37.82	14,796	10	–	6106	3.6	48.7	9.5	10	91.7
TF15	40,105	37.89	13,403	10	–	6093	2.8	55.5	9	11.7	77.6
*T. mesenterica*	40,465	28.63	6371	9	780	14,202	–	–	–	–	–

Note: The mtDNAs of TF02, TF03, TF04, TF10, TF13, and TF16 were identical, and that of TF11 and TF14 were same. Therefore information for TF04 represents that of the other five; information of TF11 represents that of TF14 in this table. Superscript 1 represents the big insertion fragment in the intergenic region; superscript 2 represents the intergenic region except for big insertion fragment. Dash means data unavailable. Mutation rates were represented by the number of single nucleotide polymorphism per one kb sequences

### Introns and other mobile fragments

**Fig. 1:**
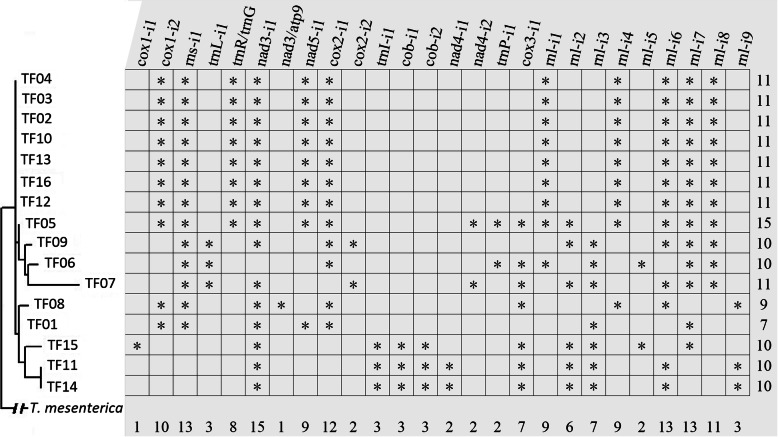
The distribution pattern of introns and big insertion fragments in the 16 *T. fuciformis* isolates. The phylogenetic tree on the left part was constructed based on the amino acid sequences of the 16 *T. fuciformis* isolates concatenated by 14 conserved protein coding genes using *T. mesenterica* as a outgroup. Stars indicate the presence of introns/big insertion fragments. The values in the last row indicate frequency of the corresponding introns/big insertion fragments found in the 16 *T. fuciformis* isolates. The values in last column represent the number of introns and big insertion fragments that the corresponding isolate contains

Twenty-four introns were identified among the 16 isolates of *T. fuciformis*, three of which were in three tRNA genes (*trnL*, *trnI*, and *trnP*), ten inserted in rRNA genes (nine from *rnl*, and the other one from *rns*), and the other eleven from seven conserved protein-coding genes (two in each of *cox1*, *cox2*, *cob* and *nad4,* one each in the other three from *nad3*, *nad5* and *cox2*) (Fig. 1). Two large mobile fragments were detected in the intergenic regions: a 1864-bp fragment located between *trnR* and *trnG* (named *trnR*/*trnG*), and a 2684-bp fragment was between *nad3* and *atp9* (named *nad3*/*atp9*). The number of introns as well as mobile fragments in each mtDNA ranged from 1 to 15. None of the introns were present in all the 16 isolates. Most mtDNAs possessed a relatively stable number of mobile elements, from 9 to 11. No mtDNA was intron-free, or harbored all the different introns.

Three introns from tRNAs were not predicted by software, but by alignment of tRNA sequences with/without introns. *trnL* gene of each isolate in the phylogenetic branch of TF06, TF07 and TF09 contained an intron, *trnL*-i1. All copies of the *trnL*-i1 showed high similarity in sequence (99.5%). Highly similar copies (99.8%) of *trnI*-i1 were detected only in the clade containing TF11, TF14 and TF15. Two *trnP-i1* copies were found in TF05 and TF06, which showed less similarity (99.1%) with 17 mismatch or indel differences. No conserved domain-encoding sequence was found in *trnL*-i1 and *trnI*-i1, but a GIY-YIG endonuclease-encoding sequence was found in *trnP*-i1.

**Fig. 2:**
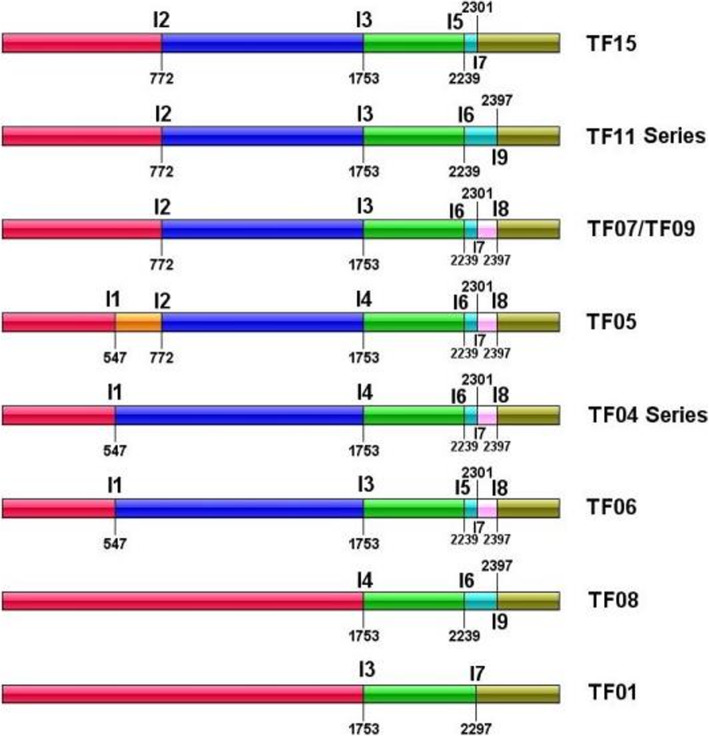
Intron landscape of *rnl* gene in the 16 *T. fuciformis* isolates. I1 to I9 represent nine introns exist in the *rnl* gene of the 16 *T. fuciformis* isolates. Boundries under/above intron names indicate the insertion sites of each intron. Number under/above the boundaries means location of introns within the *rnl* gene. TF14 shares identical *rnl* structure with TF11; TF02, TF03, TF04, TF10, TF12, TF13 and TF16 have same *rnl* gene structures

Nine introns were detected in the *rnl* gene of the 16 isolates, distributed among six insertion sites, specifically at nt 547, 772, 1753, 2239, 2301 or 2397 of *rnl* (Fig. 2). Two different introns inserted in each site at nt 1753, 2239 and 2397. *rnl*-i3 and *rnl*-i4 had same insertion site at nt 1753. *rnl*-i3 had length of 288 bp, and did not harbor genes; Whereas *rnl*-i4 was 803 bp in size, and contained a LAGLIDADG endonuclease-like ORF. The two introns showed low sequence similarity to each other. Similarly, two introns located at nt 2239 or 2397 were different from each other in length, content and sequence. Different from introns of protein-coding genes, some introns in *rnl* were small in size. Introns *rnl*-i3, *rnl*-i6, *rnl*-i7, *rnl*-i8 and *rnl*-i9 were all less than 300 bp, and did not carry any homing endonuclease genes. Tested mtDNAs were clustered into eight groups by presence/absence of these *rnl* introns (Fig. 2).

### Introns containing N-terminal duplication of the host genes

**Fig. 3:**
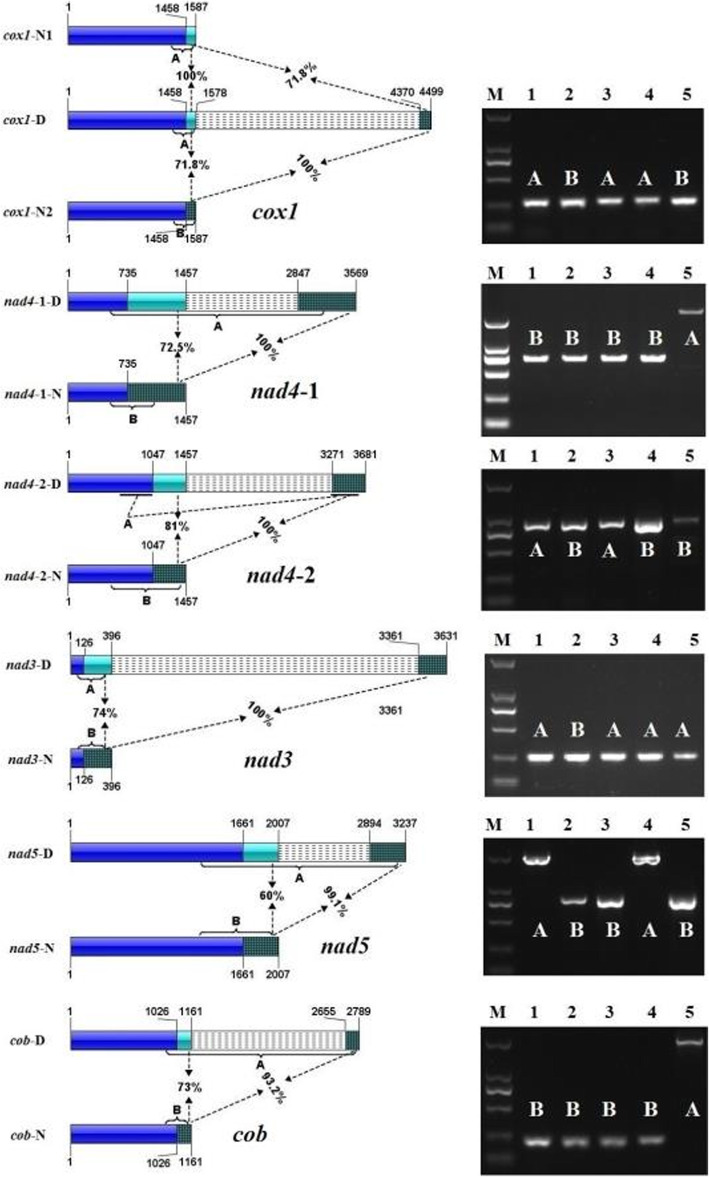
Structural comparison of *cox1* genes with / without predicted intron containing N-terminal duplication of host gene. Figures on left side: comparison of conserved genes carrying /non-carrying the predicted intron. From top to bottom were comparison diagrams of *cox1*, two for *nad4*, *nad3*, *nad5*, and *cob*. For each diagram, C-terminal of genes were represented by blue bars; N-terminal (N type), N-terminal I (D type) and N-terminal II (D type) were indicated by light green bars, which were separated by break line filled bars; bars for N-terminal II were also filled by checks. The size of each part of the gene is indicated by the number above or under the bars. Percentage indicates the amino acid identity between N-terminal (N type) and N-terminal I (D type) or N-terminal I (D type) and N-terminal II (D type). Figures on the right side: gels of PCR products for cDNAs of conserved genes the intron, which was corresponding to their left diagram (original full length ones in Supplementary Figures 1–3). Lane M indicates DNA ladder DL2000; lane 1–5 indicated products for isolates TF05, TF06, TF07, TF01 and TF11. Bands A or B were corresponding to areas pointed to by brackets A or B (or dotted line A) in the left diagram

Sequence analyses of 11 introns within protein-coding genes revealed that six had a common feature: all contained a fragment encoding an analogue of the partial host gene at 5′ end. These introns were referred to as introns with N-terminal duplication of the host genes (Fig. 3). nad4-i1 in TF11 was a 2111-bp intron, the 5’end of which showed 72.5% amino acid similarity with the following exon. *nad4*-i2 in TF05 and TF07 was a 2224-bp intron, which contained a fragment at its 5′ end showed 81% similarity with its following exon. Similarly, *cox1*-i2, *nad3*-i1, *nad5*-i1 and *cob*-i2 were introns containing their host N-terminal duplications (Fig. 3). These N-terminal duplications had similar size to, and showed high similarity with their following exons. Two different types of intron2-free *cox1* gene were detected based on downstream exon sequences (same as precursor *cox1*-N1 and exon *cox1*-N2, Fig. 3).

PCR using cDNA as template was performed to confirm the predicted introns with N-terminal duplications. Electrophoresis and Sanger sequencing results divided the six predicted introns into three types (Fig. 3): 1) *nad4*-i2 was a real intron; 2) *nad4*-i1, *nad5*-i1, and *cob*-i2 were part of the cDNA of the corresponding host genes; 3) *cox1*-i2 and *nad3*-i1 were downstream sequences of the corresponding genes.

## Discussion

### Pacbio sequencing improves short-read assemblies of *T. fuciformis*

With the rapid development of sequencing technologies and a sharp decline in the cost of whole genome sequencing, more fungal genomes have been sequenced and annotated. As an accessory of whole genome sequencing, fungal mitochondrial genomes can be assembled and identified using raw sequence data obtained [6, 9, 31, 32] based on its special characteristics, such as high copy number and a set of highly conserved genes, and then synthesized into intact molecules by PCR-based approaches. However, the presence of repetitive or non-unique DNA within mitochondrial genomes in fungi may hinder their successful de novo assembly from short reads [33]. To assess the quality of assemblies obtained from Illumina sequencing data, we generated complete mtDNAs using the Pacbio sequencing method, and aligned mitochondrial sequences from both sequencing methods of *T. fuciformis* TF13 and TF15. The differences between the two mtDNA sequences of TF13 were nine singleton indels (~ 0.022% disagreement), and for TF15 there was one singleton indel (~ 0.0025% disagreement). All indels occurred within homopolymer areas. Consistency of indels among mitochondrial genomes from different datasets (Pacbio and Illumina) of the same isolate has also been reported in *Saccharomyces cerevisiae* [8]. Sanger sequencing of these indel areas indicated that these indels resulted from sequencing or/and assembly errors using PacBio data. Thus, Illumina sequencing with 125 bp paired-end reads appeared to yield higher quality intact mitochondrial genomes for *T. fuciformis* even though the reads lengths were much shorter.

### High frequency of mitochondrial intron gain/loss in *T. fuciformis*

None of the 24 introns presented simultaneously throughout all the tested isolates. It indicates that at least one event of gain/loss took place in each of the introns after the speciation of *T. fuciformis.* Three pairs of introns, in particular, the rnl-i3 versus rnl-i4, rnl-i5 versus rnl-i6, and rnl-i8 versus rnl-i9, each pair had the same insertion site but low sequence similarity between the two introns. It means that two different introns located at the same insertion site. At least two gain/loss events took place since the speciation, in spite of the introns inserted at the same site or not. Both evidences suggest high frequency of mitochondrial intron movement among the *T. fuciformis* population.

Losses of introns are much more frequent than gains as for the spliceosomal introns in nuclear genomes [34]. Different from most nuclear introns, typical mitochondrial introns are mobile genetic elements that form self-splicing RNA molecules. The mitochondrial introns are divided into Group I and Group II according to their secondary structures and splicing mechanisms [18]. Dependent on the splicing mechanisms, introns can move either from one place to another, or even from one organism to another [18]. Taking into account the distribution pattern of introns in combination with the phylogenetic tree (Fig. 1), eight introns of the cox1-i1, trnL-i1, cox2-i2, trnI-i1, cob-i1, cob-i2, nad4-i2, and trnP-i1, are likely to gain during the population evolution of *T. fuciformis*. At least one event of intron-gain occurred at each insertion site of rnl-i3/rnl-i4, rnl-i5/rnl-i6, and rnl-i8/rnl-i9. However, no evidence supports a higher frequency of intron-loss than intron-gain in mitochondria.

### A proposed model of gene fragment exchange through gain or loss of intron with N-terminal duplication

Six introns containing N-terminal duplication were predicted from the mtDNAs of 16 *T*. *fuciformis* isolates*.* The duplications shared high similarity with exons. Each predicted intron was hypothesized to be a transposon element (TE) with host gene N-terminal homolog, which was then inserted into mtDNA of *T. fuciformis* to become an intron.

Homing reactions need three components, including 1) laterally transferred genetic elements, 2) a homing endonuclease protein, and 3) a target site [20]. Homing endonucleases with high sequence identity share homogeneous target sites [20]. It is suggested that homing reaction of the TEs (mobile intron) is performed by HE proteins they harbor, or from other places for those non-carrying HE genes. These HE genes also determine the insertion position of TEs. Speculatively, the N-terminal homologs that are just by-products of introns, may not affect the efficiency of homing reaction.

After insertion, TEs with host gene N-terminal homolog become introns of their target gene. However, PCR results of cDNAs revealed the predicted introns no longer functioned as introns for *cox1*-D, *nad3*-D, *nad4*–1-D, *nad5*-D, or *cob*-D. Predicted *nad4*-i1, *nad5*-i1, and *cob*-i2 became a part of the cDNAs. *cox1*-i2 and *nad3*-i1 as well as their following predicted exons separated from the genes, and became their downstream sequences (Fig. 3). These results indicated that different parts of D type gene may change their roles during evolution.

**Fig. 4:**
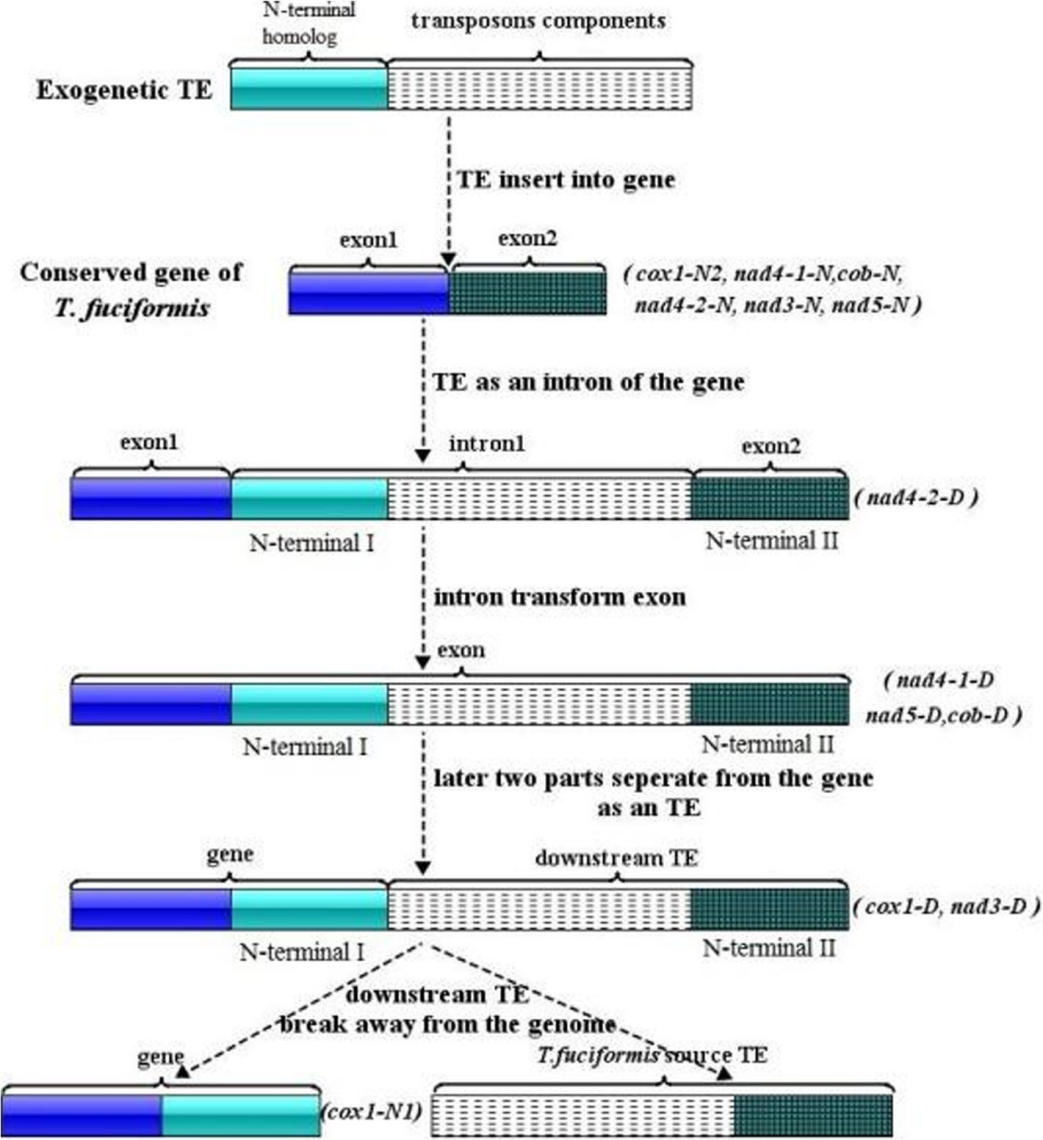
Proposed model of partial gene exchange through gain or loss of intron with N-terminal duplication of host gene. Supporting cases for each status are listed in the bracket. Exogenetic TE means gene residue precursor it carried doesn’t come from target gene

A possible model (Fig. 4) was proposed to account for the discrepancy between predicted and experimental results which the following steps: 1) a TE with exogenetic gene N-terminal homolog inserts into the conserved protein-coding gene of mtDNA in *T. fuciformis*, and becomes an intron of the gene, transforming the N type gene (*cox1*-N2, *nad4*–2-N, *nad4*–1-N, *nad3*-N, *nad5*-N, *cob*-N) into the D type gene (*nad4*–2-D); 2) the intron transforms to become a part of the exon (*nad4*–1-D, *nad5*-D, *cob*-D); 3) transposon components as well as its predicted exon separate from the gene, and become a downstream TE (*cox1*-D, *nad3*-D); 4) the downstream TE breaks away from the mitochondrial genome, transforming the D type gene into an N type gene (*cox1*-N1). The steps, including processes of insertion and differential loss of intron, result in sequence substitution of the host gene.

### Host gene fragment exchange via intron mobility is a new gene evolution approach

Lateral gene transfer refers to genetic material from a donor exchanging and stably integrating into different strains or species [35]. Previous studies on lateral gene transfer in fungi revealed that the genetic material may be individual genes, like *ToxA* [36] and *Mpk1* [37], gene clusters [38–40] and chromosomes [41, 42]. Transfer of the genetic materials import new genes or new copy of genes into host strain, which have a deep effect on disease emergence, niche specification, or shift in metabolic capabilities [43]. However, as far as we know, there is no reported evidence that fungal mitochondrial genes evolved by partial fragment exchange via lateral transfer. The above model put forward partial gene lateral transfer through gain or loss of an intron with a truncated host gene precursor, resulting in *T. fuciformis* source N-terminal of the conserved gene being replaced by an exogenetic one.

It has been assumed that the phylogenetic signal of each mtDNA gene is identical or highly similar, due to their physical locations within the same molecule [6]. However, mtDNA analyses have revealed divergence in the phylogenetic signal strength of mt genes among and within species [44]. For example, topologies inferred from concatenated *rnl* and *cox1* sequences showed significant concordance to topologies inferred from *nad4L* and *cob* among 16 isolates of *Rhizophagus* or *Glomus* species [6]. The divergence often takes place in the N-terminal, other than whole genes [6]. The above model might be a resolution for this dissimilarity: N-terminal exchange to import an exogenetic gene fragment into one gene, and greatly alter its phylogenetic signal in a single event, leading to multiple transfers during evolution that result in divergence of phylogenetic signals, where similarity is expected.

### Duplication of truncated conserved genes may be induced by introns with N-terminal homolog of host gene through horizontal gene transfer

The phenomenon with duplicated copies of conserved genes has been often found in fungal mitochondrial genomes. Large segments (more than 6 kb) were hypothesized to invert into the mtDNA of both *Phlebia radiata* [45] and *Candida albicans* [46], resulting in the duplication of *atp6* and *cox3* genes, respectively. Both inverted duplications were hypothesized to have occurred by replication-directing recombination [45, 46]. Two large inverted repeats both containing identical copies of *nad4* genes were separated by a single copy region of 5834 bp in the *Agrocybe aegerita* mitochondrial genome [47]. Duplicated sets of tRNA genes were reported in the mtDNA in *Agaricus bisporus* [16]. Duplication of the *nad4* gene in *A. aegerita* and of tRNA genes in *A. bisporus* were obtained by plasmid integration [16, 47]. Furthermore, an extra truncated *atp9* gene was found in the mtDNA of *Phialocephala subalpine* [48] and *Sclerotinia borealis* [31], and truncated *atp6* genes were detected in *Botryotinia fuckeliana* [31].

Six introns were investigated in this study and found to harbor a fragment in their 5′ end, which was a duplication of the truncated host gene, and showed high similarity with products of their subsequent exons. The length of the duplications depended on intron insertion site. If the insertion site was near the 5′ of a gene, the length of the duplication was long; if the insertion site was near the 3′ end of a gene, the length of the duplication was short. Introns with N-terminal homolog of host gene may contain fragments of other conserved genes. An extra truncated copy of the *nad2* gene was found in *cox3*-i1, located downstream of a truncated copy of the *nad3* gene. Extra truncated copies of *nad2* and *nad3* genes were always present or absent in all isolates at the same time. It is supposed that both truncated genes in *cox3*-i1 were obtained in the same way. All extra truncated genes investigated in this study were found in introns, with their coding sequences sharing high similarity with the downstream exon of host gene. The results implied that gene duplication through intron insertion is a common feature in *T. fuciformis* mitochondrial genomes.

Although occasionally, duplication of host-gene extrons in mtDNA could tend to take place more frequently near N-terminal than C-terminal. A possible explanation is that, deletion of introns together with host-gene fragments from donor mtDNA might result in loss of the fragment, while host genes near N-terminal could be more tolerant to fragment loss than those near C-terminal. In case the lost fragment locates near C-terminal, host gene would be unable to transcribe, leading to a loss of gene function. However, host gene would still be able to transcribe, completely or partially, when the lost fragment locates near N-terminal. Consequently, host-gene segments carried by introns were observed to concentrate near N-terminal, and then insertion of the introns into recipient mtDNAs leads to host-gene duplications near N-terminal.

### Annotation errors without intra-specific comparisons

Conserved protein-coding genes and rRNAs in fungal mitochondrial genome were annotated by the MFannot [11, 49] or BLAST [50] programs; their intron-exon boundaries were identified by Clustal W by comparison with intron-free homologous genes of closely related species [50]; and tRNAs were identified by MFannot [49], tRNAscan-SE [51], RNAweasel, and/or Rfam [32]. However, because of the great differences existing among intergenic regions of interspecific mitochondrial genomes, some annotation errors might have occurred in these alignments. These errors were reflected mainly in the annotations of introns with N-terminal homolog of host gene, and introns within tRNAs. In this study, six introns with truncated host gene precursor were not detected by MFannot, but by alignment with the corresponding intron-free genes of intra-specific isolates. The common feature of these introns was that they contained a fragment at the 5′ ends, which was a ‘duplication’ of their following exon. As a result, the software could not identify the real exons. RNAweasel, Mfannot, and tRNA-SE were used to identify tRNAs in *T. fuciformis*, and no intron containing tRNA was found. However, three tRNAs with introns were identified among 16 mitochondrial genomes by intra-specific comparison, which were *trnL* in TF06, TF07, and TF09, *trnI* in TF11, TF14, and TF15, and *trnP* in TF05, and TF06. The short sequence length of tRNAs made it difficult for programs to annotate the introns they carried. High similarity of sequences not only in conserved genes but also intergenic regions among intra-specific mtDNAs made intron-insertion boundaries clearer. Intra-specific mitochondrial genome comparison improved quality of their gene annotation.

## Conclusions

In the study, we analyzed 16 mitochondrial genomes of *Tremella fuciformis*. Intraspecific mitochondrial genomic comparison revealed that coding sequences, tRNA, and rRNA genes were conserved, whereas introns and intergenic regions were variable. In total, 24 introns were detected inserted in protein-coding genes, rRNAs and tRNA genes, and intron number varied greatly between isolates. Sequence comparison revealed six instances where introns harbored N-terminal duplication of the host gene. This implied that N-terminal duplication originated from external sources (foreign organisms), and might replace the corresponding exon of host gene. Our findings also revealed intron mobility as one of the reasons for duplication of truncated conserved genes in fungal mitogenomes and the phenomenon of introns with N-terminal duplications makes fungal mitogenome annotation even more difficult with more attention needed to properly define the components.

## Methods

### Fungal isolates and DNA extraction

Sixteen *T. fuciformis* isolates (TF01-TF16) were obtained by the Edible Fungal Germplasm Resources Management Center of Fujian province, Fuzhou, China. The origin of the isolates is listed in Supplementary Table 3. Among them, TF15 was isolated from Wuyishan National Parks, Fujian, China, in 2014, TF11 and TF14 were obtained from Wuyishan National Nature Reserve in 2015, and TF01 was another wild isolate from Huboliao National Nature Reserve of Fujian.

After being grown on potato dextrose broth at 25 °C for 48 h, single yeast-like cells of *T. fuciformis* were washed and harvested by centrifugation at 10,000 g for 5 min, and stored at − 20 °C after freeze-drying. For Illumina sequencing, total genomic DNA of 16 *T. fuciformis* isolates was extracted using the Omega HP Plant DNA Kit according to the manufacturer’s instructions; at least 500 ng DNA (> 18 ng/ul) was required for each sample. For PacBio sequencing, single molecule real-time (SMRT) sequencing, long DNA fragments of TF02 and TF15 were isolated using the cetyl trimethylammonium bromide (CTAB) method as described in www.pacb.com/wp-content/uploads/2015/09/ DNA-extraction-chlamy-CTAB-JGI.pdf; at least 20 μg DNA (OD260/280 between 1.8 and 2.0, OD260/230 between 2.0 and 2.2, intact gDNA > 20 kb) was required for each sample.

### Genome sequencing, assembly, and gene annotation

Whole genome shotgun sequencing of 16 *T. fuciformis* isolates was performed at Beijing Novogene Bioinformatics Technology Co., Ltd. using the Illumina HiSeq 2500 platform with paired-end libraries, targeting 3–6 Gb data per isolate. The raw Illumina sequencing data of *T. mesenterica* ATCC28783 (accession SRX8046622) was downloaded from the SRA database of NCBI. Raw reads were assembled using Velvet 1.2.03 [52].

Mitochondrial contigs were identified by BLAST against published mitochondrial genome of *Cryptococcus neoformans* var. grubii H99 (accession NC_004336). Mitochondrial contigs were extended step by step according to the pair-end relationship of reads: if one read mapped on end of a contig, the other end may extend the sequence. Ambiguous extensions or gaps were confirmed or closed by PCR sequencing. Contigs were concatenated into single circular DNA sequences based on 100% overlap.

PacBio sequencing technology was used to verify the assembly accuracy of two of the Illumina-sequenced isolates, TF13 and TF15. These were sequenced using PacBio RS II, targeting approximately 2.5 Gb raw data per isolate. Genome assembly for PacBio sequencing data was done using the Canu 1.3 program [53]. Single contigs for each mitogenome were identified by comparison with mitochondrial genomes of the corresponding isolates obtained from Illumina sequencing data, to obtain complete circular DNAs after trimming 3′ ends.

Both gene prediction and gene annotation were initially done using the online tool MFannot (http://megasun.bch.umontreal.ca/cgi-bin/mfannot/mfannotInterface.pl). tRNAs were annotated by combining the results of MFannot, tRNAscan-SE [54], and RNAweasel [55]. Conserved gene boundaries and exon-intron junction points were confirmed by comparison with corresponding intron-free genes of other tested isolates using Clustal X [56].

### Phylogenetic analysis of *T. fuciformis* isolates

To determine the evolutionary relationships among the 16 *T. fuciformis* isolates, concatenated amino acid sequences of 14 conserved genes (*atp6*, *atp8*, *atp9*, *cob*, *cox1*, *cox2*, *cox3*, *nad1*, *nad2*, *nad3*, *nad4*, *nad4L*, *nad5*, and *nad6*) totalling 4252 characters, were used for phylogenetic analysis, using *T. mesenterica* as an outgroup. Amino acid alignments were done using Clustal W in the MEGA 6 program [57] with gap opening penalty and gap extensive penalty values of 10 and 3, respectively (same as pairwise and multiple alignments). A phylogenetic tree was constructed using Maximum Likelihood in MEGA 6, and tested by Booststrap analysis with 500 replications. Gaps and missing data within alignments were treated as deletions.

### PCR analysis to confirm special predicted introns

PCR analyses were used to confirm predicted introns. Primers (Supplementary Table 4) were designed using online tool primer-blast from NCBI website. These primers targeted regions of cDNA from upstream exon to N-terminal sequence, and in special cases, regions from upstream exon to N-terminal duplication. Representative isolates were selected for the PCR work; mtDNAs of these isolates had to include all of the introns, and corresponding intron-free sequences.

Yeast cells were collected at logarithmic phase, and RNA was extracted using the Omega HP Plant RNA Kit. cDNA was reverse transcribed using the PrimeScript™ RT-PCR Kit (Takara, Dalian), and used as PCR templates. PCR products were sequenced at Sangon Biotech (Shanghai).

## Supplementary information

**Additional file 1: Supplementary Table 1.** Sequencing and assembly statistics of 16 isolates.

**Additional file 2: Supplementary Table 2**. Length and GC content of different areas in 16 isoaltes of T. fuciformis as well as T. mesenterica ATCC28783.

**Additional file 3: Supplementary Table 3**. The source of isolates used in this study.

**Additional file 4: Supplementary Table 4.** PCR primers for confirming cDNA sequences of D type genes.

**Additional file 5: Supplementary Figure 1.** Original full length gel image for *nad*5, *cob*, *nad*4–2 and *cox*1 (sub image R5, R6, R3 and R1, respectively) in Fig. 3. Lane M indicates DNA ladder DL2000; lane 1–5 indicated products for isolates TF05, TF06, TF07, TF01 and TF11. **Supplementary Figure 2.** Original full length gel image corresponding to sub image R2 (gel image of *nad*4–1) in Fig. 3. Lane M indicates DNA ladder DL2000; lane 1–5 indicated products for isolates TF05, TF06, TF07, TF01 and TF11. **Supplementary Figure 3.** Original full length gel image corresponding to sub image R4 (gel image of *nad*3) in Fig. 3. Lane M indicates DNA ladder DL2000; lane 1–5 indicated products for isolates TF05, TF06, TF07, TF01 and TF11.

## Data Availability

All differentiated mtDNAs of *T. fuciformis* in the study are deposited in GenBank, accession numbers of which are MF422647 (TF01), MF422648 (TF04), MF422649 (TF05), MF422650 (TF06), MF422651 (TF07), MF422652 (TF08), MF422653 (TF09), MF422654 (TF11), MF422655 (TF12), and MF422656 (TF15), respectively. Both raw sequencing data of *T. mesenterica* ATCC28783 (accession SRX8046622) and mtDNA sequence of *Cryptococcus neoformans* var. grubii H99 (accession NC_004336) are available in NCBI website.

## Abbreviations

mtDNA: Mitochondrial DNA
rRNA: Ribosomal RNA
tRNA: Transfer RNA
IEP: Intron-Encoded Protein
*atp*6, 8, and 9: ATP synthase F0 subunit 6, 8, and 9
*cob*: Cytochrome b
*nad*1, 2, 3, 4, 4 L, 5, and 6: NADH dehydrogenase subunit 1, 2, 3, 4, 4 L, 5, and 6
*cox*1, 2, and 3: Cytochrome c oxidase subunit I, II, and III.
cDNA: Complementary DNA
TE: Transposon Element
CTAB: Cetyl Trimethylammonium Bromide
