# Supplementary material for: Intra-specific comparison of mitochondrial genomes reveals host gene fragment exchange via intron mobility in Tremella fuciformis
Source: BMC Genomics. 2020 Jun 24;21:426. doi: 10.1186/s12864-020-06846-x (PMC7315562; doi:10.1186/s12864-020-06846-x)
Supplement: Supplementary file 3 — Additional file 3: Supplementary Table 3. The source of isolates used in this study. [file 12864_2020_6846_MOESM3_ESM.docx]

Supplementary Table 3 The source of isolates used in this study.

| Isolate No. | Source |
| --- | --- |
| TF01 | Wild strain obtained from Huboliao National Nature Reserve, Zhangzhou, China in 2011 |
| TF02 | Strain T0019 |
| TF03 | Strain T0026 |
| TF04 | Cultivar T0033 obtained from Sanming Fungal Institute |
| TF05 | Cultivar T0042 isolated from Zhangzhou city |
| TF06 | *Tremella fuciformis* strain 704 isolated by Sanming Fungal Iinstitute |
| TF07 | *Tremella fuciformis* strain 707 isolated by Sanming Fungal Institute |
| TF08 | Wild strain obtained from Nanjing, Zhangzhou by Xinghua fungal Institute |
| TF09 | Wild strain obtained from Wuping, Longyuan in 2015 |
| TF10 | Wild strain 1^#^ isolated by [Asia University, Taiwan](http://www.baidu.com/link?url=QbbpOVIgx1jJSsevXvWeixSCDssWfCShmRQg3rzBfK_2wqVYEJmn67Isu_9f8eFa) |
| TF11 | Wild strain 2^#^ isolated from Wuyishan National Nature Reserve in 2015 |
| TF12 | Cultivar T0081 |
| TF13 | Tr26, monokaryon of cultivar T0035; T0035 is a strain divided from Tr21 from 1970s-1990s. |
| TF14 | Wild strain 1^#^ isolated from Wuyishan National Nature Reserve in 2015 |
| TF15 | Wild strain isolated from Wuyishan National Parks in 2014 |
| TF16 | Wild strain 2^#^ isolated by Asia University, Taiwan |
