# Supplementary material for: Intra-specific comparison of mitochondrial genomes reveals host gene fragment exchange via intron mobility in Tremella fuciformis
Source: BMC Genomics. 2020 Jun 24;21:426. doi: 10.1186/s12864-020-06846-x (PMC7315562; doi:10.1186/s12864-020-06846-x)
Supplement: Supplementary file 4 — Additional file 4: Supplementary Table 4. PCR primers for confirming cDNA sequences of D type genes. [file 12864_2020_6846_MOESM4_ESM.docx]

Supplementary Table 4. PCR primers for confirming cDNA sequences of D type genes.

| Primer ID | Sequences | Product type |
| --- | --- | --- |
| cox1_F | TGGACTTGCAGGTATGCCAC | A or B |
| cox1_R | ATGCGTGGAATGGTGTAGGA |  |
| nad4-1-1F | GTGGCTGGTTCGGTACTGTT | A |
| nad4-1-1R | GCTCCGAAAGCTAGTCGGTTA |  |
| nad4-1-2F | GGAGAATCACCCGCTAGTGT | B |
| nad4-1-2R | TAGCATCTGGCATGAACCCA |  |
| nad4-2-F | TGGGAATTTGGGGTGGTTCA | A or B |
| nad4-2-R | GCTCCGAAAGCTAGTCGGTTA |  |
| nad3_F | GCTCATCGTCCAGATGCAGAA | A or B |
| nad3_R | AAGAGCACCTTTACCTAACTCGT |  |
| nad5_F | CACGGTATGGCTGACCAACA | A or B |
| nad5_R | AGCTACTGATCGTCCTGCATT |  |
| cob_F | TCGAGGTAGCCAATTCCGAC | A or B |
| cob_R | TGTTGCAATGTCCATCAGTGTG |  |
