# Supplementary material for: Intra-specific comparison of mitochondrial genomes reveals host gene fragment exchange via intron mobility in Tremella fuciformis
Source: BMC Genomics. 2020 Jun 24;21:426. doi: 10.1186/s12864-020-06846-x (PMC7315562; doi:10.1186/s12864-020-06846-x)
Supplement: Supplementary file 5 — Additional file 5: Supplementary Figure 1. Original full length gel image for nad5, cob, nad4–2 and cox1 (sub image R5, R6, R3 and R1, respectively) in Fig. 3. Lane M indicates DNA ladder DL2000; lane 1–5 indicated products for isolates TF05, TF06, TF07, TF01 and TF11. Supplementary Figure 2. Original full length gel image corresponding to sub image R2 (gel image of nad4–1) in Fig. 3. Lane M indicates DNA ladder DL2000; lane 1–5 indicated products for isolates TF05, TF06, TF07, TF01 and TF11. Supplementary Figure 3. Original full length gel image corresponding to sub image R4 (gel image of nad3) in Fig. 3. Lane M indicates DNA ladder DL2000; lane 1–5 indicated products for isolates TF05, TF06, TF07, TF01 and TF11. [file 12864_2020_6846_MOESM5_ESM.docx]

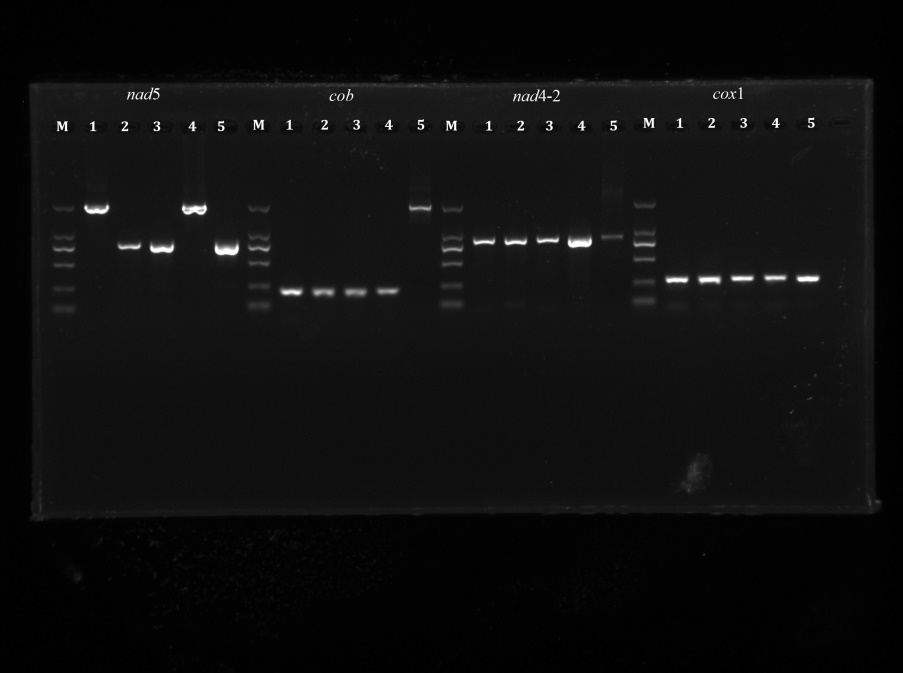


Supplementary Figure 1: Original full length gel image for *nad*5, *cob*, *nad*4-2 and *cox*1 (sub image R5, R6, R3 and R1, respectively) in Figure 3. Lane M indicates DNA ladder DL2000; lane 1-5 indicated products for isolates TF05, TF06, TF07, TF01 and TF11.


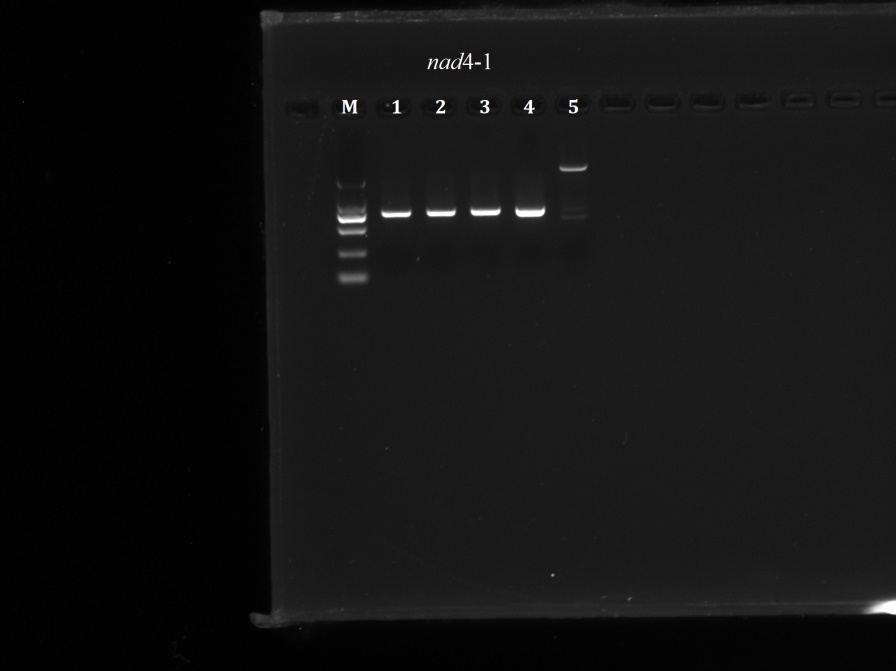


Supplementary Figure 2: Original full length gel image corresponding to sub image R2 (gel image of *nad*4-1) in Figure 3. Lane M indicates DNA ladder DL2000; lane 1-5 indicated products for isolates TF05, TF06, TF07, TF01 and TF11.


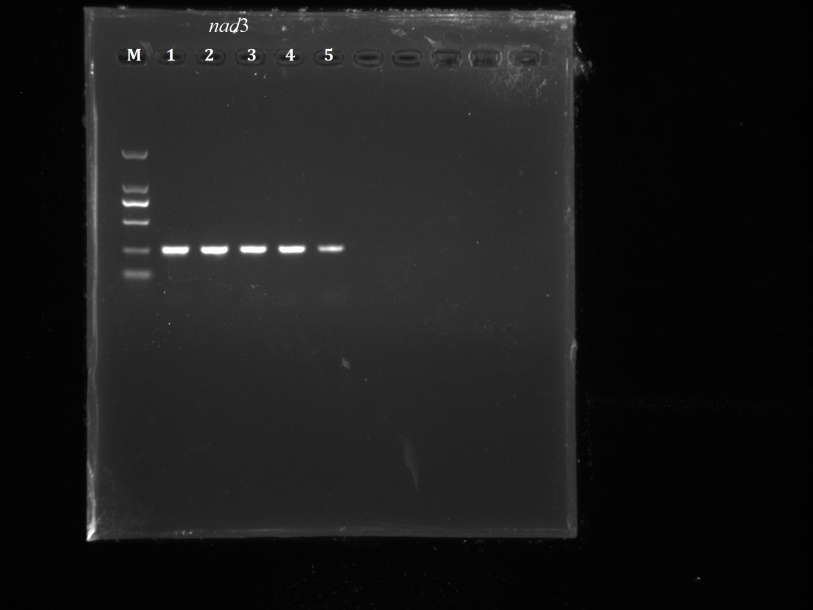


Supplementary Figure 3: Original full length gel image corresponding to sub image R4 (gel image of *nad*3) in Figure 3. Lane M indicates DNA ladder DL2000; lane 1-5 indicated products for isolates TF05, TF06, TF07, TF01 and TF11.
